# Adaptation of the Foot and Ankle Physical Exam for Telehealth

**DOI:** 10.1177/1556331620974675

**Published:** 2021-02-21

**Authors:** Oliver B. Hansen, Stephanie K. Eble, Scott J. Ellis, Mark C. Drakos

**Affiliations:** 1 Hospital for Special Surgery, New York, NY, USA; 2 Geisel School of Medicine, Dartmouth College, Hanover, NH, USA

**Keywords:** virtual physical examination, telehealth, foot and ankle examination, telemedicine

## Introduction

The COVID-19 pandemic has restricted the practicality of in-person visits, as hospitals seek to reduce capacity in order to maintain social distancing for patients and staff. Under these novel circumstances, healthcare providers have been forced to rely on telehealth visits to care for patients with nonurgent conditions. Even as restrictions begin to lift in many hospitals, patient preferences seem to indicate that telehealth will continue to play a large role once the pandemic subsides. While previous studies have found telehealth to be effective, its use in orthopedics was limited prior to the COVID-19 pandemic[1,2,7,8,10]. This is, to some degree, a reflection of the belief that a meaningful physical exam cannot be performed virtually. Since orthopedists view the physical exam as central to diagnosis, this notion has served as a barrier to the broader adoption of telehealth in the field.

While these concerns are legitimate, our experiences over the past months have demonstrated that a thorough physical exam can indeed be performed virtually for the foot and ankle. Though technical and physical barriers to an exhaustive exam remain, many conditions can be appropriately triaged through this modality. We thus present a set of guidelines for performing a virtual foot ankle physical exam. Finally, we include a discussion of the benefits of telehealth, as well as its current limitations.

## Virtual Foot and Ankle Physical Exam

After describing patient preparation, we will outline a core exam, including visual inspection and assessment of range of motion, strength, and neurovascular irregularities. We will then go on to describe special tests that can be conducted at the examiner’s discretion. These include tests for flat and cavovarus foot deformities, hallux rigidus, and Achilles tendon injuries.

### Preparing for the Telehealth Visit

The foot and ankle exam can be made more efficient and effective if patients are prepared beforehand. Each patient should fill out forms describing their chief complaint, current illness history and symptoms, and general medical history, including medications, allergies, and social history. Patients should also provide all available vital signs, including height, weight, heart rate, temperature if possible, and blood pressure if available. These data can improve the documentation required for billing and performing a high-level exam.

**Fig. 1.**
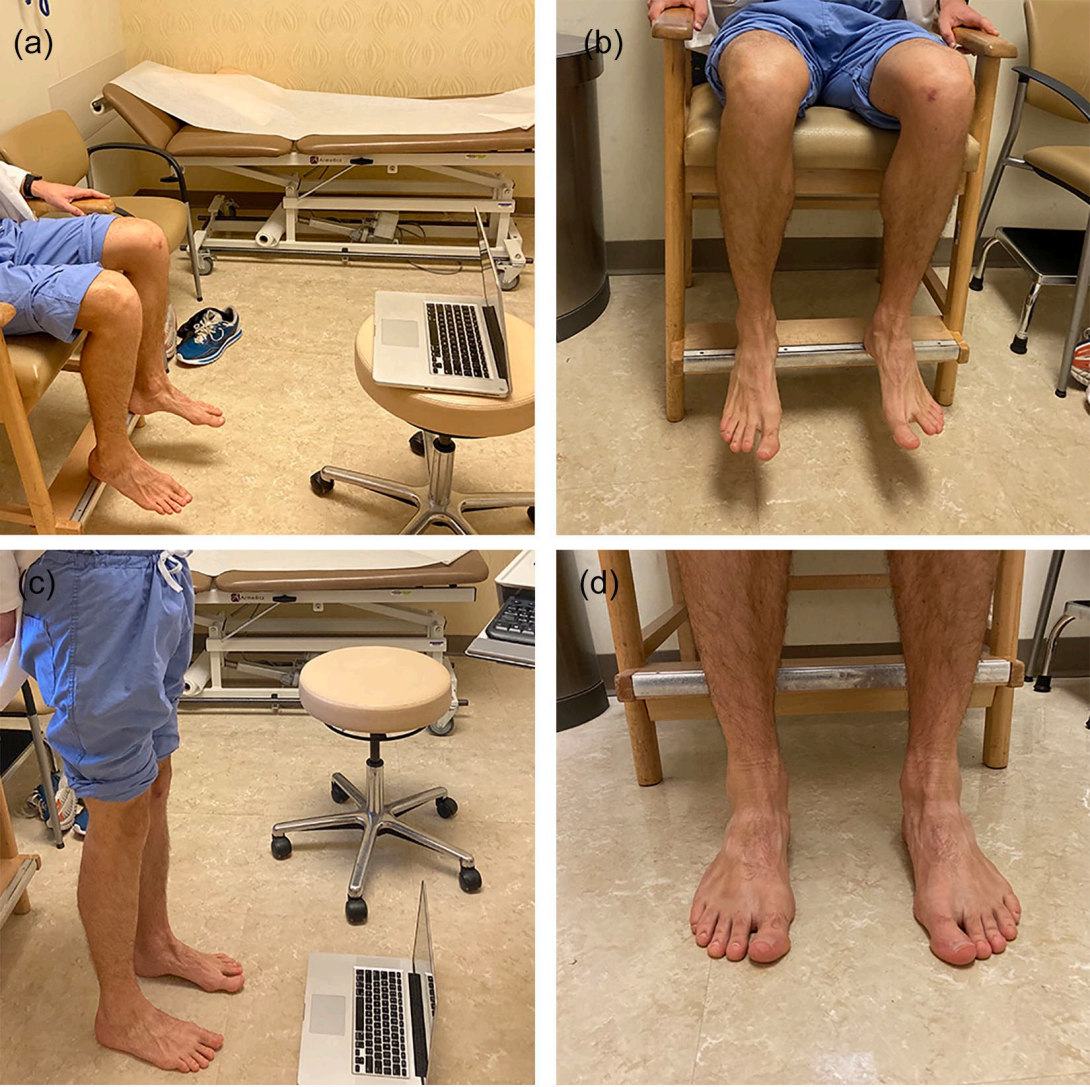
The patient will reorient the camera and place it at roughly shin level to allow the provider to see the feet and ankles: (a) Computer setup, (b) desired framing for seated view, (c) computer setup, and (d) desired framing for standing view.

The patient’s telehealth appointment will be most effective if they adhere to the following guidelines for setup and attire. We recommend that the patient use a laptop or tablet for the exam, as these are both stable and portable. This allows the camera to be adjusted as needed during the exam. Patients will ideally need 10 to 15 feet of open space in front of the camera so that the clinician can examine their gait. They should also seek to orient the camera away from any light sources, such as light fixtures or windows, for optimal visibility. The patient should test their camera and microphone prior to the appointment, as well as the various camera angles described. We find this can expedite the visit considerably, as camera setup will vary depending on the patient’s device. The visit will start with the camera at eye level, and the patient will eventually reposition the camera so that the feet and ankles are visible, according to the provider’s instructions (Fig. 1).

When conducting the virtual exam, clinicians may benefit from the use of a checklist that more carefully details each component of the exam and allows for thorough documentation of results [4].

### Core Exam

#### Gait analysis

The clinician can assess the patient’s gait by asking them to walk away from the camera for at least 4 steps before turning around and walking back. This will allow the provider to analyze ankle alignment during movement as well as knee and ankle flexibility. The patient can repeat this while walking on their toes and heels in order to assess their ability to dorsiflex and plantarflex the ankle above the gravity threshold.

#### Inspection and palpation

A general visual assessment can be performed with the patient sitting or standing in front of the camera before turning around and holding the camera over the tops of their feet. Alignment can be reexamined in a stationary position during this time. The provider can also look for atrophy, deformity, prior incisions or scars, erythema, and rash. The patient should hold the plantar aspect of the foot so that it faces the camera, at which point it can be assessed for ulcers or skin pathologies. The provider should ask the patient to point to the physical structures that are causing pain or discomfort, while ensuring that these parts are visible on camera. Fortunately, with respect to the foot and ankle, most structures are subcutaneous and direct palpation often has a high correlation with only 1 or 2 possible anatomic locations that may be causing symptoms.

#### Joint motion

The provider should assess both active and passive range of motion of the ankle and hindfoot joints, and if relevant the metatarsophalangeal joints. These can also be observed while the patient is walking, but may be more closely monitored with the patient sitting in front of the camera. For passive range of motion, ideally a friend or family member will manipulate the joint while the patient resists the urge to tense any muscles. The provider should ask the patient whether moving the joint through the full range of motion causes pain, and if so in which part the pain is most pronounced. This process can be performed for ankle plantarflexion and dorsiflexion with a bent knee, and hindfoot inversion and eversion. To evaluate gastrocnemius tightness, the patient should repeat the ankle plantarflexion and dorsiflexion motion with a straightened knee. This range of motion can then be compared to their previous range with a bent knee.

#### Strength tests

Most strength tests will require the patient to have an assistant present, and even then strength remains difficult to accurately determine remotely. The provider can determine the patient’s ankle plantarflexion strength by their ability to walk on their toes, which indicates at least 4/5 plantarflexion strength. The tests outlined in Table 1 can be performed with a friend or family member acting as the examiner. The examiner should clearly communicate perceived strength and any asymmetries directly to the care provider.

**Table 1.** Descriptions of strength tests that can be performed by an examiner in order to assess absolute strength and asymmetries for a variety of movements.

Strength test	Instructions
Ankle dorsiflexion	The examiner places their hands on the tops of the patient’s feet. The examiner resists as the patient pulls their feet upward, toward their shin.
Ankle plantarflexion	The examiner places their hands against the balls of the patient’s feet. The examiner resists as the patient pushes their toes down, as if pressing the gas pedal.
Big toe extension	The examiner places their hands on the top of the patient’s great toes and resists as the patient pulls their toes upward. The ankle should remain still, with just the toe moving.
Eversion	The examiner places their hands on the outside of the patient’s feet and pushes in. The patient resists this motion while attempting to keep the ankle in a neutral position.
Inversion	The examiner places their hands on the inside of the patient’s feet, along the arch, and pushes out. The patient resists this motion and attempts to keep the feet steady.

#### Circulation

While pulses cannot be assessed by the care provider, it is still possible to visually assess perfusion and capillary refill during the virtual exam. The provider can ask the patient to show both sides of the foot to the camera, then can ask if both feet feel the same temperature to the patient’s touch, or ideally to an assistant’s touch if available. The patient can then press the pad of the big toe until it turns white, and allow it to return to pink while on camera. The provider should note how long this takes. Pitting edema can also be assessed when the patient presses their shin with 2 fingers, just above the ankle. Calf pain, such as that which might signify a deep vein thrombosis (DVT), can be assessed by asking the patient to squeeze the calf. If a DVT is suspected, the patient should receive an ultrasound.

#### Neuromuscular

Sensation can be assessed by asking the patient to touch different parts of the foot and ankle. If a friend or family member can do the touching instead, this may yield more reliable results. First, the provider can ask if the patient is experiencing numbness or tingling and to point to the area. The provider can then assess the function of individual nerves by asking the patient or their companion to touch the following areas. For the superficial peroneal nerve, touch the top of both feet within the first dorsal webspace. For the deep peroneal nerve, touch the webspace between the big toe and second toe. For the tibial nerve, touch the bottom center of the foot. For the sural nerve, touch the outside of both feet. For the saphenous nerve, touch the inside of both calves. The care provider should ask the patient if sensation is the same on both legs when assessing each of these nerves.

This concludes the core portion of the foot and ankle exam. The following special tests can be performed if they are relevant based on suspected pathology.

### Special Tests

The tests outlined in this section can help the provider virtually diagnose a ruptured Achilles tendon, hallux rigidus, flatfoot, or cavovarus foot. This is not an exhaustive list, but rather serves as an example of some of the conditions that may effectively be assessed during a telehealth exam.

#### Ruptured Achilles

If the patient has sustained an Achilles injury, the Thompson test can be performed while the provider watches. This will require the patient to have an assistant present. The patient should lie face down, preferably on a couch or bed so that their feet are hanging off the end. The camera should be positioned so that the ankle is visible, and the assistant will then squeeze the calf while the patient relaxes their muscles completely. The degree of ankle plantarflexion upon squeezing can be compared to the opposite leg. If lying on the floor, the patient can bend their knee to an angle of 90°.

#### Hallux rigidus

The provider can assess range of motion at the first metatarsophalangeal joint if hallux rigidus is suspected. With the forefoot in view of the camera, the patient should actively move the big toe through its full range of motion. The patient will then use their fingers to move the toe to assess passive range of motion. While it will be challenging to quantitatively assess range of motion by video, the provider should ask the patient to describe any symptoms such as pain, grinding, or catching that accompany this movement.

#### Flatfoot

The patient should position the camera so that their lower legs are visible and then face away from the camera. They can perform heel raises on both legs, followed by each leg separately while touching a surface for balance. The provider should watch for inversion during these movements to assess posterior tibial tendon function. They can also assess overall alignment based on the presence of the “too many toes” sign.

#### Cavovarus foot

A book or stack of magazines can be used to perform a Coleman block test for patients with a cavovarus deformity. Once the camera is positioned so that the heel is visible, the patient will place their heel on the block as well as the lateral portion of their foot. Their first, second, and third toes should hang over the edge. The clinician will assess the degree of hindfoot correction in this position.

### Postoperative Follow-Up

Some patients may prefer to use telehealth for follow-up after surgery due to geographic constraints or travel restrictions. The postoperative exam can incorporate elements of the core exam where relevant, though weight-bearing portions may not be feasible depending on the patient’s status. The provider should be sure to get a clear and well-lit view of the wound in order to monitor healing progress and check for drainage, swelling, or rash around the incision site. Virtual follow-up will be insufficient in some cases, and the exam’s central purpose is to monitor issues that could require an in-person visit. Due to the limitations of the telehealth exam, the clinician should not hesitate to ask the patient to schedule an in-person visit if issues regarding would healing or other complications do arise.

## Discussion

Many of the elements of a standard physical exam can be performed virtually, with some adaptation as we have illustrated thus far. This physical exam can be further supplemented by imaging studies, which the patient can perform locally and upload for the clinician’s reference. However, we do not see the virtual foot and ankle exam as a true replacement to an in-person appointment, but rather as a screening tool or backup option for circumstances that make an office visit challenging. It is currently impossible to definitively comment on outcomes after a virtual exam. Resistance to telehealth in orthopedics means that adoption has been slow and thus the currently available literature is limited. We expect that our understanding of telehealth’s role in orthopedics will progress rapidly given its increased use during the COVID-19 pandemic.

### Time and Efficiency

Recent studies have shown that telehealth can offer orthopedic patients increased efficiency and good outcomes. For example, a randomized controlled study compared virtual and conventional follow-up visits for orthopedic trauma [8]. Patients receiving telehealth follow-up had shorter visits and did not have to miss work, while over half of the patients with in-person follow-up did miss work. It should be noted that this study involved a small sample to begin with, and roughly one quarter of patients dropped out in each group. Retrospective evidence has also shown that telehealth visits are associated with shorter wait times in orthopedic and sports medicine populations [2,12]. The reduction of travel time and expenses will further improve the efficiency of a telehealth visit for patients. Clinicians, on the other hand, will likely need to dedicate more time to preparation for a telehealth visit and thus spend more overall time per visit [8,12]. This has been consistent with our own experience. We have found that, when new patients upload all of their radiologic exams and complete forms ahead of the appointment, a routine visit will take approximately 15 minutes. Follow-up and postoperative visits routinely take approximately 5 to 10 minutes, depending on patient familiarity with the computer setup and the complexity of the case.

Telehealth also has the potential to save resources, both for the patient and the healthcare system as a whole [2,10]. More research will be needed to confirm these findings, but with lower wait times, decreased cost, and lack of travel time, telehealth may often be more convenient for patients. This notion is evidenced by consistently high levels of patient satisfaction [2,8,10,11]. However, these benefits must be taken into account alongside the increased time burden that telehealth places on clinicians.

### Postoperative Use

While the exam we have described may function as an initial screening visit, other authors have found telehealth to work well for postoperative visits [1,5,9]. Findings from a postoperative telehealth visit seem to largely agree with in-person findings after certain knee and upper-extremity procedures [1,5,13]. This suggests that telehealth can recognize complications that need attention, and while we expect that results would be similar for a foot and ankle exam, further study will be needed to confirm this. Telehealth follow-up may be better suited for later visits, as during the visits immediately following surgery patients may need suture or cast removal. In 1 study, patients did attempt to remove their own sutures during a telehealth visit after carpal tunnel release [13]. Only 10 of 16 patients were able to successfully take out their sutures, and the authors identified this as the biggest challenge to a virtual postoperative visit. We thus imagine that the virtual exam may be well suited to routine follow-up visits for patients with a thoroughly healed wound who have resumed weight bearing without complaints.

### Technological Limitations

Telehealth faces additional technological limitations because it requires an Internet connection, appropriate device, and some degree of fluency with the technology. While 1 study reported high rates of satisfaction with audio and video quality during a telehealth visit [8], some patients may lack a computer or tablet equipped with a front-facing camera. Elderly patients in particular may feel less comfortable with video conferencing and camera manipulation. In randomized trials that have assigned patients to telehealth visits, 10% to 20% have opted out, citing preference for an in-person visit [6,8]. Virtual exams may also feel impersonal compared to an in-person visit, and thus may not allow the patient and clinician to develop a sense of trust and understanding. However, such factors are difficult to quantify and 1 study found that patients were largely satisfied with the level of personal connection during telehealth visits with a range of specialists [3]. While this study did have a large sample size, it only included established patients and it might be more difficult to establish a personal connection with a new patient over telehealth.

### Future of Telehealth

We expect that demand for telehealth will remain elevated beyond the COVID-19 pandemic, as patients recognize the convenience offered by a telehealth exam. For new patients, however, we view the exam primarily as a screening tool. It may be especially helpful in reaching patients who do not live near a foot and ankle specialist, thus increasing access to appropriate care for these patients. Experienced clinicians have developed a keen tactile sense that can increase the accuracy of diagnosis compared to a patient’s self-assessment of strength, stiffness, or other physical characteristics. For this reason, in-person evaluation may be a logical next step for new telehealth patients who may benefit from further care. While COVID-19 mandated a rapid, wholesale adoption of telehealth at many hospitals, we see it occupying a narrow role for foot and ankle orthopedics in the immediate wake of the pandemic.

That role may grow as improved technology becomes more commonly available and people gain competency in its use. We can imagine telehealth benefiting patients in exciting ways, such as by allowing multiple care providers to conference with a patient simultaneously. For example, this might involve a patient meeting with an orthopedic surgeon and physical therapist simultaneously to develop a comprehensive and individualized treatment plan. Telehealth could also allow patients to quickly reach specialized physicians in the setting of an acute injury via their mobile phone. Telehealth in its current form faces limitations for the foot and ankle exam, but this technology does carry the power to expand patients’ access to receiving care from orthopedic specialists.

A thorough physical foot and ankle exam can be performed virtually, though it does require some modifications of the in-person exam and may be time-intensive for the clinician. This exam can be used as a screening tool for new patients seeking treatment from a foot and ankle specialist or potentially to streamline routine follow-up visits for the patient. A virtual exam does not replace in-person assessment. Clinicians must be conscious of its limitations in making certain diagnoses and should carefully consider whether further imaging modalities or an in-person visit are needed for thorough evaluation.

## Supplemental Material

sj-zip-1-hss-10.1177_1556331620974675 – Supplemental material for Adaptation of the Foot and Ankle Physical Exam for Telehealth Click here for additional data file. Supplemental material, sj-zip-1-hss-10.1177_1556331620974675 for Adaptation of the Foot and Ankle Physical Exam for Telehealth by Samuel A. Taylor, Joseph D. Lamplot, Oliver B. Hansen, Stephanie K. Eble, Scott J. Ellis and Mark C. Drakos in HSS Journal®: The Musculoskeletal Journal of Hospital for Special Surgery
